# Fatherhood and addictive disorders: experiences and support needs – results of a qualitative interview study

**DOI:** 10.3389/fpsyt.2026.1867867

**Published:** 2026-07-06

**Authors:** Christoph Beineke, Thomas Altenhöner, Fabian Pioch, Janina Dyba, Thorsten Köhler

**Affiliations:** 1 Faculty of Social Sciences, Hochschule Bielefeld – University of Applied Sciences and Arts, Bielefeld, Germany; 2 School of Social Work, University of Applied Sciences and Arts Northwestern Switzerland (FHNW), Olten, Switzerland; 3 Fachverband Sucht+ e. V., Bonn, Germany; 4 Faculty of Social Services, Catholic University of Applied Sciences of North Rhine-Westphalia, Cologne, Germany

**Keywords:** abstinence, family, gambling disorder, gaming disorder, paternal, substance use disorder

## Abstract

**Objectives:**

Parental addictive disorders are a major public health problem. Despite already known negative effects, the parental role of men seeking help is rarely acknowledged within addiction support services. This study aims to identify existing relations between fatherhood and addiction, and the resulting support needs of affected fathers.

**Methods:**

For data collection 15 fathers with addictive disorders were interviewed by using qualitative, guided interviews. The data material was analyzed using qualitative content analysis.

**Results:**

The effects of addictive disorders on fatherhood are predominantly unfavorable. Conversely, fatherhood can have consumption-reducing as well as consumption-increasing effects. In this context, five topics were identified in which fathers expressed specific support needs. Particularly fathers with substance use disorders expressed their need for further support, while fathers with behavioral addictions expressed a lesser need.

**Conclusions:**

Paying attention to fatherhood in the context of addiction services provides a starting point for increasing the motivation to change. Individual support needs should be considered when implementing father-specific programs. Not only the fathers themselves, but also their children can benefit from successful treatment and expected changes in fatherhood.

## Introduction

From a global perspective, drug use is increasing, with men being more affected than women (1). In Germany, addictive disorders are among the most common mental illnesses (2). In 2024, the 30-day prevalence of alcohol consumption among men was 73% (women: 64%), the 12-month prevalence of cannabis use was 12% (women: 7.1%) and that of illicit drugs amounts to 4.6% (women: 2.7%) (3). Consequently, the prevalence of substance use disorders (SUD) was also higher among men (3). In 2025, the prevalence of gambling disorder was 3.2% among the male adult population, and 6.9% showed high-risk gambling behavior (women: 1.0%; 4.1%) (4).

However, there is a lack of valid data on the number of affected fathers and co-affected children. In German addiction treatment services in 2024, around 26% of men in outpatient or inpatient treatment stated that they had an underage child. In many of the cases they did not live with their child (5). Further estimates assumed that between 5.1-9.2% of underage children in Germany lived in the same household as an adult with alcohol use disorder, while around 0.6-1.2% of children experienced adults with drug use disorder in the same household (6). Parental gambling disorder affected 4.0-4.2% of minor children in Germany, most of whom lived with their parents (7). Due to the problematic psychosocial impact, not only on the individual with an addictive disorder, but also on their family members, parental addiction poses a particular public health problem (8, 9). Parental SUD and behavioral addictions (BA) are known to have negative effects on children. Specifically, those children have been identified as being at increased risk of developing an addictive or other mental disorder of their own (10–12).

Frequent physical or emotional absence has been reported for fathers with SUD (13–16). When contact exists, the quality of the father-child relationship is often impaired and characterized by tension and avoidance (15, 17, 18). A lack of warmth, attention, empathy, and affection towards the child is frequently observed, as a result of substance use (15, 19). The likelihood of insecure attachment is increased (19, 20), and a lower relationship quality is common (21, 22). SUD are also associated with reduced parenting skills and other related problems. Notable are increased tendencies towards harsh disciplining and the use of violence (15, 23), as well as lenient reactions (24, 25) or erratic parenting behavior (25, 26). Parents also show lower monitoring skills and less knowledge about their child (13, 27, 28). Parental gambling disorders are also associated with reduced parenting skills (22).

Despite these findings, addiction research and treatment has so far focused primarily on affected mothers, while fatherhood-related issues are neglected (15, 29, 30). Only a few studies exist on the subjective experience of fatherhood in this context. These describe a comparatively lower level of parental satisfaction and effectiveness (21, 31). Some data regarding fathers with SUD show that they are often aware of the negative effects of their disorder and describe a perceived failure to fulfil their role as fathers, possibly leading to feelings of guilt and shame (13, 14, 16, 32, 33). Affected fathers experience an incongruence between the expectations they place on their parental role and how they actually fulfil it (13, 15, 16). Even if fathers are not present in the children’s lives, fatherhood can have an emotional relevance. Feelings of loss and failure are important in this context (32). These negative emotions, feelings of being overwhelmed, or prohibited contact with their child can trigger an increase in substance use or relapses (15, 34), so that consumption can be seen as an attempt to regulate and cope with this negative and even painful emotions. Especially when negative emotional states predominate and positive emotions are lacking, there is a risk for developing dysfunctional emotion regulation strategies to counterbalance these negative states, e.g. through substance use, gambling or gaming (35). Difficulties in emotion regulation can be linked to consumption and addictive disorders (36, 37) due to short-term pleasant effects on personal mood and experiences (35). Thus, it is conceivable that individuals who already suffer from addictive disorders may also respond to stressful events related to fatherhood by using or increasing their use. However, these dynamics can again aggravate issues linked to fatherhood and parenting, which can potentially lead to renewed negative emotions. On the other hand, fatherhood is described as a strong motivator to strive for changes, whereby the desire to become a more involved father is essential (13, 15, 32, 33). At the same time, this is perceived as a great challenge and source of pressure (32). Thus, affected men must deal with consumption-related as well as fatherhood-related demands in their everyday lives. Despite the existing connections, these are hardly considered in addiction treatment. Due to limited attention, there is a need to further analyze how affected men experience their fatherhood and how specific support can be designed (38).

## Materials and methods

### Research questions

Considering the limited data, the situation of affected fathers needs to be analyzed in depth, so interventions tailored to the needs of the target group can be developed. The following research questions were explored in the context of this qualitative interview study:

1. How does SUD/BA affect fatherhood?
2. How does fatherhood affect substance use/gambling/gaming?
3. Which fatherhood-related support needs express affected fathers?

### Study site

The interview study is part of a research project funded by the German Federal Ministry of Health. The project cooperated with ten outpatient and inpatient addiction support facilities in North Rhine-Westphalia (federal state of Germany).

### Participants and recruitment

Men who were considered eligible were approached by the staff of the cooperating treatment facilities and informed about the study. Men who were interested in participating were screened for inclusion in the study and invited to participate, if they met following inclusion criteria:

- biological or social fatherhood
- problematic use/addictive disorder (alcohol, illicit drugs, gambling or gaming)
- Consumption within the past twelve months

Fathers with other psychiatric disorders, primarily in need of treatment, were excluded.

The selection of participants was based on theoretical considerations, according to which fathers with SUD and BA as well as fathers in and not in contact with their children were included. As an incentive, the participants received a 25€ voucher.

### Data collection methods and analysis

Data collection was carried out using semi-structured individual interviews following a problem-centered approach. The interviews were based on thematic sections and questions, which were developed prior to the interviews based on the current state of research and to which the participants give their answers. Thus, the guideline focused on specific topics that structured the interviews and enable comparison (39). Sociodemographic data were recorded on a supplementary sheet.

The interviews took place on the premises of the addiction support facilities. Staff members were not present. The interviews were realized in German, recorded on dictation devices, and subsequently transcribed. The first author is a social worker and doctoral student. He worked as a research assistant on the research project and conducted the interviews.

The data was analyzed using the software MAXQDA24 in accordance with a structuring, qualitative content analysis (40). The structuring content analysis is based on a deductive category system, which was developed prior to the analysis, based on existing scientific findings and the research questions. It also included a brief definition for every category and, if necessary, a distinction from other categories. However, when analyzing the data, it was possible to add inductive categories if appropriate. The interview material was structured and analyzed based on these categories. A second research assistant reviewed several coded transcripts, and potential differences were discussed to refine the category system and category definitions. The results presented are limited to the questions posed in this paper.

### Ethical considerations

The study was approved by the Ethics Committee of the Catholic University of Applied Sciences of North Rhine-Westphalia. Written informed consent was obtained from all participating fathers and this consent may be revoked with future effect. In accordance with the project’s data protection guidelines, only authorized members of the research team had access to the collected data.

Prior to the interview, participants were informed that the data would be analyzed and used only in anonymized form. If names, cities, or institutions were mentioned during the interviews, they were subsequently redacted.

Participants were assured, that their participation was independent of the addiction treatment and non-participation would not have negative effects. Additionally, there was the possibility to skip single questions, pause the interview, or exit it without providing a reason or experience negative consequences. If any indications of potential child protection concerns had arisen during the interview, they would have been discussed with the principal investigator to determine how to proceed.

Fathers with addictive disorders can be seen as a vulnerable target group due to their mental illness and the interview participation or some questions may result in negative emotions and negative effects of the treatment process. To minimize any potential negative effects, we excluded fathers with other psychiatric disorders, primarily in need of treatment, and we worked with the staff to recruit interviewees. Furthermore, as described above, participation was voluntary, participants were informed about the content and objectives of the study, and had the option to refuse participation or stop the interview at any time.

### Researcher reflexivity

The authors were four men and one woman in different ages and with different academic backgrounds (social work, public health, psychology, social sciences). Two authors were fathers themselves. The first author was a social worker with practical experience in child and youth services and research assistant. The second author was a public health expert specializing in prevention and health promotion. The third author was a social worker and research assistant. The fourth author was a psychologist and research assistant with extensive research expertise in the field of family and SUD. The fifth author was a social scientist specializing in research methods. All authors were interested in addiction and prevention research, but had different levels of knowledge in this research area.

During the research process, a deliberate shift in perspective was intended to focus on the fathers, their lived experiences and support needs, although the living conditions of children affected by parental addictive disorders cannot be ignored. The findings were therefore discussed in light of the current state of research to determine how fathers with addictive disorders can be supported in their paternal roles so that family members can also benefit. The involved researchers were aware, that personal experiences, the own father role and ideals about fatherhood may influence interpretations. Research team discussions as well as discussions of results with other persons (e.g., addiction counselors, other researchers) provided an opportunity to reflect on the results.

## Results

### Participants

From July 2023 to February 2024, 15 interviews were conducted, which lasted between 33 and 83 minutes (M(SD)=59.9(14.3) min.). The demographic characteristics of the sample are shown in **Table 1**.

**Table 1:** Sample characteristics, self-reported.

Participant	Age	Main problem	Current use status	Comorbidity	Living with child’s mother	Number (age) of children	Contact with children
F01_gd	26	BA	abstinent	–	yes	1 (1)	same household
F02_gd	39	BA	abstinent	–	yes	1 (6)	same household
F03_gd	52	BA	abstinent	–	yes	1 (18, not biological)	same household
F04_d	58	illicit drugs: heroin, cannabis, alcohol	abstinent	–	no	4 (37; 22; 21; 14)	regularly
F05_gd	39	BA	abstinent	depression, burnout	yes	2 (4; 2)	same household
F06_a	49	alcohol	abstinent, but still cannabis	–	no	1 (16)	same household
F07_d	26	illicit drugs: cocaine, gambling; other illicit drugs in the past	abstinent	depression	no	1 (4)	no contact for ten months
F08_d	36	illicit drugs: methamphetamine	abstinent	–	no	1 (11)	regularly
F09_a	40	alcohol; in the past also illicit drugs	abstinent	depression, anxiety	no	1 (17)	same household
F10_gd	34	BA	abstinent	–	yes	1 (1)	same household
F11_gd	39	BA	abstinent	–	no/yes^a^	3 (9; 5; 1)	same household
F12_d	39	illicit drugs: cannabis	abstinent	depression	no	1 (5)	regularly
F13_a	29	alcohol; in the past also illicit drugs	abstinent	depression	no/yes^b^	2 (11, biological; 9, not biological)	no contact for nine years with biological child; regular contact with new partner’s child
F14_d	34	illicit drugs	abstinent	depression, borderline personality disorder, sleep disorder, ADHD	no	1 (16)	never
F15_a	54	alcohol	abstinent	depression	no	3 (30; 25; 19)	no contact for one year

^a^ First child with another mother; Father now lives with his second wife and all three children.

^b^ No contact with the mother of his biological child; Father lives with his new partner who has a child.

The participants were between 26 and 58 years old (M(SD)=39.6(9.8) years) and had between one and four children (M(SD)=1.6(1.0) children). While the proportions of fathers with alcohol-related, drug-related and gambling-related main problems were similar, only one father with gaming disorder was interviewed. As a result of the recruitment strategy, all fathers were actually using addiction support services when the interview took place. All participants with BA were recruited in the same outpatient addiction support facility. Four of the five fathers, who used illicit drugs, were recruited in one inpatient facility and the fifth in an outpatient facility. With regard to the fathers, who used alcohol, two were recruited in an outpatient facility and two in an inpatient facility.

Two fathers with current alcohol use disorder also used illicit drugs in the past and one father with drug use disorder also gambled, when he used drugs. At the time the interviews were conducted, all participants were abstinent, which can be interpreted in light of the ongoing addiction treatment. Only one father who sought help due to his alcohol consumption was still using cannabis without intention to change. On the contrary, he highlighted positive effects of cannabis use to calm down and fight alcohol craving. Seven fathers reported comorbid mental disorders, with depression reported in all seven cases and additional disorders reported in three cases (burnout; anxiety; borderline personality disorder, sleep disorder, ADHD). Noteworthy, the majority of fathers with SUD, but only one father with BA reported comorbid mental disorders.

With regard to living arrangements with the child’s mother and the child, differences were also observed in the sample between fathers with SUD and BA. All fathers with BA lived with the child’s/children’s mother, only one father was divorced from his first wife and living with his new partner. In contrast, no father with a SUD lived with the child’s mother. Only one father had a new partner who already had a child of her own, for whom he took on a father role, but he lived separated from his biological child. All fathers with BA and two single-parenting fathers with alcohol-related problems reported living with their children. Four fathers had no contact with their biological children, but one of them had contact with the child of his new partner. The remaining three fathers reported regular contact with the child in consultation with the child’s mother.

### Relations between fatherhood and consumption

Besides the impact of consumption on fatherhood, which was predominantly negative, almost all fathers described changes in their substance use/gambling/gaming behavior that they directly attributed to their role as fathers. **Table 2** summarizes the described relations between fatherhood and consumption.

**Table 2:** Perceived relations between fatherhood and consumption.

	Effects of consumption on fatherhood	Effects of fatherhood on consumption
Negative	- Poor mood (e.g., irritability, restlessness, impatience) - Limited temporal/emotional availability - Withdrawal from the parental role - Lack of awareness of children‘s needs and negative effects - Distancing by the children - Conflictual relationship with the child‘s mother, loss of contact with the child	Consumption-enhancing aspects: - Conflicts with child‘s mother - Loss of contact with the child - Family demands - Changes in the couple relationships after the birth
Positive	+ Better interaction with the child + More time spent together	Consumption-reducing aspects: + Structuring effects of birth, new demands and tasks + Situational abstinence during visits + Long-term motivation to change, living fatherhood as a goal + Desire to establish/maintain contact with the child

### Impact of consumption on fatherhood

The impact of paternal SUD/BA on the relationship with the child was perceived in different ways. Some of the fathers described how their disorder had little to no influence on the father-child relationship. Crucial factors for this perception were abstinence during interactions with the child or the assessment that the child did not witness the use. However, several fathers reported that they only recognized impacts after achieving abstinence:

“My son has of course noticed it before. What I didn’t realize is that he was aware of it.” (F09_a)

If effects on the relationship were noticed, these were particularly evident in the deterioration of the fathers’ mood (e.g., irritability, lack of attention, restlessness, impatience, bad mood). In addition, the time spent with the child and emotional availability was limited by the procurement and use of drugs or gambling/gaming. As a result, the fathers described a low level of motivation for shared activities and a retreat from their roles as caregivers. Some fathers reported that their children became increasingly distant or noticed changes in the family dynamics that they found difficult to interpret. One father described how his child had behaved more caringly in times he was in a poorer mental state. Four fathers with SUD lost contact with their children, primarily due to their substance use.

When children found out about the use, they reacted in various rejecting ways according to the interviewed fathers. Some children expressed the wish that their fathers would use less, while others expressed unease and a loss of trust. In one case, there was a complete loss of contact after the discovery. In contrast, one father reported that his child, after an initial shock, was proud of him for seeking treatment.

Only few fathers also described positive effects of the substance use on the relationship. For example, one father spent more time with his child while using drugs:

“Yes, he probably didn’t even notice. Of course, he thought it was great, dad is there for him then, we play together then.” (F09_a)

After achieving abstinence, some of the fathers experienced improvements in the father-child relationship. In their perception, their children noticed the end of negative effects too:

“…I think that she definitely noticed it. [ … ] But now that I am abstinent again, it is just the way it is, because the joy and the sense that something can be experienced together are there.” (F08_d)

### Impact of fatherhood on consumption

#### Reduction of use

For several fathers, the birth of their child was a crucial point in time for reducing their use:

“…but when my daughter was born, I had an abstinent phase of two and a half years for the first time, without therapy.” (F08_d)

The deciding factors for the changes were the feeling of responsibility and the fear of being unable to function as a father. Three fathers described a lifestyle change, characterized not only by reduced substance use, but also by taking up employment or distancing from criminal milieus. Men encountered new responsibilities, challenges and distractions through becoming a father. These changes supported their attempts to reduce their use. Single-parenting fathers described particularly clearly, that they see themselves in a better position to fulfil the responsibilities associated with fatherhood, after a use reduction.

Direct contact with the child also constituted an important ‘regulator’ for paternal use. Most fathers described the attempt to be abstinent during visitations:

“However, when I was visiting my daughter or she was staying with me, I didn’t use at all.” (F13_a)

Although childbirth and contact with their children prompted the fathers to temporarily reduce their use, they did not indicate a lasting behavioral shift. Contacts with the child may be associated with a situational abstinence, but some fathers also described compensatory effects or no effects at all:

“Drug-use actually remained unchanged, I must say [ … ]. So the amount was always the same and I usually made up for it when I couldn’t use the other day.” (F12_d).

In addition, some fathers reported shifts in their substance use, focusing on certain substances (alcohol, cannabis), but never a complete abstinence.

Fathers with BA likewise saw being with their children as a key motivation for staying free of gambling/gaming and thus being available for their children:

“…wife and kids. I was just afraid of losing them because of it. And I believe I just need to think about losing them and I immediately know I would never want to gamble again.” (F11_gd)

Several fathers reported threats of separation by their partners if they did not change their patterns of consumption. Therefore, they focused on achieving abstinence to maintain their relationships. Taking on childcare responsibilities and spending time together can have a beneficial effect and support efforts to stay abstinent.

In addition to situational changes, the interviewed fathers described long-term attempts to reach abstinence. Based on their own perceptions of ideal fatherhood, they strived for an abstinent future, especially if the current form of parenting was not in line with their ideals:

“…because I always ask myself, what kind of role model do I want to be for my child? And there, abstinence is extremely important.” (F07_d)

In the long-term perspective, the desire to implement and/or maintain contact with the child appeared to be central. For fathers without contact, their fatherhood was a strong motivator, since achieving abstinence was of central importance for the intended realization of fatherhood. Disruptions in contact were associated with grief, longing and a feeling of guilt. However, some fathers also showed signs of acceptance of the situation and primarily wanted to focus on their treatment:

“To truly accept that I can’t influence this at the moment, and that I don’t want to either, because I want to take care of myself first.” (F07_d)

Once they had achieved abstinence and structured their everyday life, the fathers wished to resume the contact with their children. By contrast, one father wanted to see his children right away and, based on the conviction that he had destroyed the relationship through his SUD, expressed the wish to redeem himself.

#### Intensification of use

Fatherhood can also contribute to increased use. Some fathers described a link between their consumption and family stress. They mentioned the demands of caring for a child in connection to a stressful job, and changes in the couple’s relationship after the birth of their child. However, conflicts with the child’s mother and the loss of contact with the children were mentioned more often as factors that trigger stress, grief and negative emotions, and thus increased use.

The relationship with the child’s mother was often conflictual due to consumption. All nine fathers with SUD lived separated from the child’s mother, and the relationships were often characterized by problems that continued even after separation. In some cases, mothers were found to have restricted father-child contact because of such conflicts. Due to a lack of alternative coping strategies, conflicts may lead to increased consumption:

“Well, the child’s mother was a constant stressor, she was often enough the trigger. If I’m being honest.” (F06_a)

Fathers with BA mostly described their relationship with the child’s mother as harmonious. However, the revelation of their disorder represented a turning point in the relationship and led to a loss of trust. Several fathers experienced an attempt at social control in their relationship, e.g., in relation to their bank accounts or threats of separation if they did not seek addiction treatment. Unlike in the context of SUD, this pressure led to concrete attempts to change rather than to increased use.

In addition to longer breaks in parental contact with their child, periods of intermittent contact were also described. Reduced contact with the child was accompanied by increased use:

“Because I was cut off from my child, my use increased because it was very stressful for me at the beginning.” (F14_d)

Reduced or non-existent contact was often caused by the separation from and/or conflicts with the child’s mother. However, one father described how he was more involved in new relationships, and another father reported having completely avoided reaching out to spare his child the uncertainty of irregular contact. Due to interruptions in contact, the fathers missed developmental steps of their children and felt hindered in building or maintaining a good relationship with them. They reacted to these negative experiences by increasing their use, while at the same time, use often triggered conflicts.

#### Fatherhood-related needs

Based on the interview data, five central topics can be identified in which the interviewees reported needs that arose in the context of their fatherhood connected to their disorder. These are illustrated and described in **Table 3** and include ‘general fatherhood issues’, ‘fatherhood in the context of addiction’, ‘legal issues’, ‘parenting’ and ‘support for father-child communication’.

**Table 3:** Described fatherhood-related support needs.

Topic	Expressed needs	Exemplary interview statements
*General fatherhood issues*	- Low-threshold counselling services - Clarification of paperwork related to fatherhood - Masculinity-related aspects (e.g., ideas of masculinity, acknowledging feelings, taking a break)	- *“A point of contact for men, but what is also publicly known.”* (F09_a) - *“It's hardly ever talked about. It's always, you know, men have to be strong here and there. And men don't have any problems. They can handle anything.”* (F12_d)
*Fatherhood in the context of addiction*	- Addressing fatherhood within the context of addiction treatment - Reflecting on fatherhood and the father-child relationship - Dealing with negative feelings such as feeling overwhelmed or guilty - Personal handling of conflictual situations with the child's mother or with contact bans - Exchange with other affected fathers	- *“Yes, maybe something like, why do I use when I know my child is sleeping next door? What is the reason for my use? Is it because of all the stress? Or is it because I want to take some time out? [ … ] Or what about when my child is sad and I don't know how to reach them? And perhaps I often blame myself.”* (F13_a) - *“If there really were such groups, it would definitely help a lot of people. Yes. Simply to be able to share and to know, there's someone else who has the same problems as me. I think that would give you strength. Definitely.”* (F12_d)
*Legal issues*	- Rights as a biological father - Options for establishing contact - Custody rights - Sources of further support	- *“Yes, what options do I have as a father? Should I take legal action? As a father, what am I legally entitled to? What rights do I have? Yes, something like that.”* (F07_d)
*Parenting*	- Learning parenting skills, helping to prepare for life with a child - Exchanging ideas with other fathers about parenting	- *“Maybe education in certain things, how to deal with them. I mean, I taught myself everything [ … ], or what I took from my parents or from my sisters. But I actually jumped into the deep end on my own.”* (F09_a)
*Support for father-child communication* - *Addressing the issue of paternal use* - *Addressing the issue of (potential) child use*	- Instructions for starting and structuring a conversation - Age-appropriate education about SUD/BA and treatment - Dealing with rejection from the child	- *“But maybe I wished I had had some kind of guideline back then on how to approach something like this most effectively. [ … ] It is a very sensitive topic, after all, and it's not that easy for a father to admit such a weakness to his son.”* (F06_a)
	- Dealing with personal fears that the child will start using drugs in the future or develop a SUD - Dealing with the situation if the child actually starts using drugs - Instructions for starting and structuring a conversation - Sources of further support	- *“Yes, maybe somehow the fear that you have as a father that your children might take the same path as you did, [ … ] because there is no guideline for that. So, there is no manual for that. Maybe that's a bit what's generally overlooked. The focus is put on the addict. But, yes, that's perhaps one aspect of how to deal with the addiction and the fear that the children will take the same path as you as a parent, in this case as a father.”* (F06_a)

The above-mentioned needs were expressed primarily by fathers with SUD. Only two gambling fathers spoke of difficulties in explaining their disorder to their children, but for the most part, these fathers saw no direct connection between fatherhood and their BA and were convinced that they would be able to fulfil their role as fathers once they had achieved abstinence:

“…I believe that if I no longer gamble [ … ], I will actually become a better father because I will have a clearer head and more time.” (F02_gd)

## Discussion

Based on semi-structured interviews with affected fathers, the associations between SUD/BA and fatherhood were examined. On this basis, the study explores fatherhood-related support needs, which should be considered within addiction treatment. There were numerous relations between fatherhood and SUD/BA, which were perceived by fathers to varying degrees. The effects of SUD/BA on fatherhood were particularly evident in the unfavorable influence on the father-child relationship, which manifested in a poorer mood and a lower emotional and temporal availability. In very few cases, a positive influence on the father-child relationship was reported, although this should be viewed critically given that the fathers were not always aware of the consequences of intoxication. Despite possible positive influences, negative effects – such as a stronger focus on consumption and neglecting paternal duties – seemed to predominate in the long term.

On the other hand, fatherhood also influenced consumption. In this context, both consumption-enhancing and consumption-reducing aspects can be identified. In the given sample, in particular birth and (intended) contact with the child, in connection with one’s ideal of an involved fatherhood, had a consumption-reducing effect, while conflicts with the child’s mother and contact bans enhanced the use. In this context, consumption can be seen as an individual coping strategy, which, however, can contribute to increased or further problems (41) and maintenance of the addictive disorder (35). In addition to addressing the fatherhood-related needs mentioned in the interviews to support fathers in fulfilling their paternal role, it seems also necessary to enhance emotion regulation skills in order to cope with challenging emotional effects that can arise from fatherhood.

In some areas, relevant differences were found between fathers with SUD and BA. All fathers with SUD lived separated from the child’s mother and, with two exceptions, also from their children. In some cases, there was no contact with their child and the majority reported psychiatric comorbidities. In contrast, fathers with BA in the presented sample showed less stress (mostly no psychiatric disorders, living with the child’s mother, ongoing contact with the child). Furthermore, in the context of SUD, intoxication can be associated with an increased risk of physical violence or negative parenting (32), as well as changes in behavior that are incomprehensible to children (9). The different personal circumstances of fathers with SUD and BA in the given sample may therefore have led to individual support needs. Five topics were identified in which affected fathers expressed the need for further support. While fathers with BA hardly saw any need for in-depth engagement with their fatherhood, the need for support was extensively expressed by fathers with SUD. It is conceivable that, due to a higher burden of problems, parenting was less successful and negative, or missing parenting experiences led to greater uncertainties regarding their role as father. In a quantitative survey, opioid-dependent fathers in current methadone treatment showed more negative assessments of their fatherhood and lower satisfaction with parenting compared to non-dependent fathers (31). However, a quantitative survey of fathers with gaming disorder also suggests a connection to lower parental self-efficacy expectations (21). In addition, negative effects on children can also be expected from a father’s BA (12, 22). In the conducted interviews, the fathers with BA described adverse effects on emotional availability and mood, although they were convinced that they would overcome these difficulties once they achieved abstinence. Follow-up surveys would be necessary to determine whether further support is needed.

However, it should be noted that not only addictive disorders, but mental disorders in general are associated with difficulties in parenting, such as feelings of failure to fulfil a successful parental role, unfavorable parent-child interactions and strained relationships, from which further support needs arise (42). In the final sample of this interview study, in particular fathers with SUD had comorbid mental disorders, which could lead to more comprehensive difficulties. It is not possible to clearly distinguish between the effects of SUD and those of comorbid mental disorders on parenting and it can be assumed that not only the addictive disorder, but also the overall mental health conditions shaped the fatherhood-related difficulties. During the treatment of addictive disorders and discussing fatherhood, these potential connections should also be taken into account.

It can be concluded that treating paternal addiction is essential to ensure a positive environment for children. Fathers who were aware of adverse effects on their fatherhood described a high motivation to change. A combination of individual and group therapy can be useful to address the expressed support needs, but the necessity contrasts with the scarcity of father-specific services within the addiction support system. Therefore, the development of father-specific interventions is required (15, 30, 43). Since fathers described numerous connections between fatherhood and consumption, which have also been reported in other studies (13, 15, 32, 33), this topic can serve as a starting point for further changes. Prochaska et al. describe the process of behavioral changes through the cyclical sequence of the stages of *precontemplation, contemplation, preparation, action* and *maintenance* (44). The interview statements seem to be particularly relevant to the stages of *contemplation* and *maintenance*. Given the personal significance, fatherhood can lead to intentions to initiate behavioral change. In addition, a strong connection between intended change and one’s self-identity, as well as knowing of the effects on the social environment, can support the transition to *action* (44). By addressing these aspects, it may be possible to increase the willingness and commitment to treatment. After achieving abstinence, fatherhood should be considered in context of relapse prevention. *Maintenance* is particularly successful when triggers for relapses are recognized and alternative coping strategies are learned (44). This requires a detailed understanding of the stressors that arise from fatherhood and consideration of the support needs expressed by fathers. Additionally, fathers need the necessary skills to organize contact with the child’s mother and the child, especially communication, parenting and coping skills. It seems sensible to offer parental interventions not only after, but also during addiction treatment (45). These interventions should be tailored to the knowledge, skills and individual needs of the participants. To meet these comprehensive issues, cooperation with other institutions (e.g., men’s counselling, legal advice, child protection services) appears to be necessary. Even after achieving abstinence, fathers may still need support to fulfil their father role. It is therefore important that they know where to turn for further help. Therefore, institutional networks between addiction and family support services are needed (15, 24).

Due to the circumstances (e.g., voluntary participation), it was not possible to reach additional fathers within the project timeline. Among fathers with SUD and gambling disorder, the most recent interviews did not yield any substantial new findings. Nevertheless, there is a certain methodological weakness – which entails limitations – in the composition of the sample that was reached. In future studies, specific subgroups of fathers with different addictive disorders should therefore be examined separately.

## Limitations

It can be assumed that the interviewees tended to be highly motivated in the context of fatherhood. Fathers who attach little personal importance to their fatherhood were presumably less willing to take part in an interview. In addition, only fathers who already make use of addiction services were approached. It is likely that this group differs from fathers who do not (yet) seek help. Furthermore, the fathers were approached by the staff in the institutions. Although advertising material was available, the addiction counselors were significantly involved in the recruitment. Lastly, fathers with gaming disorder were hardly reached, so that no statements can be made about this subgroup.

## Conclusion

Despite some limitations, it became very clear from the interviews that there are numerous interactions between fatherhood and SUD/BA. Besides seeing fatherhood as a driving force for abstinence, fatherhood-related stressors must also be considered to implement functional coping strategies. It is therefore necessary to address the support needs expressed by fathers to create specific interventions aimed exclusively at affected fathers. However, fatherhood should also be considered as a general topic in individual counselling. As a result, addressing fatherhood could have a positive influence on the addiction treatment.

Achieving abstinence does not automatically lead to successful fatherhood, but it can be a first and important step (13). Additionally, this would not only benefit the fathers themselves, but also their children, providing an opportunity to prevent transgenerational transmission of addictive disorders.

## Data Availability

The raw data supporting the conclusions of this article will be made available by the authors, without undue reservation.
